# Anesthetic management of laparoscopy-assisted total proctocolectomy in a cardiac sarcoidosis patient with a cardiac resynchronization therapy-defibrillator: a case report

**DOI:** 10.1186/s40981-020-00350-7

**Published:** 2020-06-06

**Authors:** Yutaro Kammura, Ai Fujita, Yuji Karashima, Shoko Nakayama, Kazuhiro Shirozu, Tadashi Kandabashi, Ken Yamaura

**Affiliations:** 1 Department of Anesthesiology and Critical Care Medicine, Kyushu University Hospital, Fukuoka, Japan; 2 Operating Rooms, Kyushu University Hospital, Maidashi 3-1-1, Higashi-ku, Fukuoka, 812-8582 Japan; 3 Department of Anesthesiology and Critical Care Medicine, Graduate School of Medical Sciences, Kyushu University, Fukuoka, Japan; 4 Medical information Center, Kyushu University Hospital, Fukuoka, Japan

**Keywords:** Cardiac sarcoidosis, CRT-D, Anesthetic management

## Abstract

**Background:**

Cardiac sarcoidosis (CS) causes severe conduction abnormalities and arrhythmias. CS patients are increasingly being treated with cardiac resynchronization therapy-defibrillators (CRT-Ds). For the first time, we report the anesthetic management of a CS patient with a CRT-D.

**Case presentation:**

A 65-year-old male with an implanted CRT-D due to CS was scheduled for a laparoscopy-assisted total proctocolectomy for his transverse colon cancer. His left ventricular ejection fraction was 32.0%, and his physical status was a New York Heart Association class III. General and epidural anesthesia were performed while using standard monitors and a FloTrac^TM^ system. The dual-chamber pacing (DDD) modality of the CRT-D was unchanged, and its defibrillation function was deactivated before surgery. The surgery was successfully performed, and the patient was discharged without worsening of his cardiac condition.

**Conclusions:**

A detailed understanding of this patient’s condition, as well as sarcoidosis, helped to facilitate successful anesthetic management of this patient.

## Background

Sarcoidosis is a multisystem disorder of unknown etiology characterized by a non-necrotizing granulomatous inflammation [1]. Symptomatic cardiac involvement is reported in approximately 5% of patients with sarcoidosis [2, 3]. The three main manifestations of cardiac sarcoidosis (CS) are conduction abnormalities; ventricular arrhythmias, including sudden cardiac death; and heart failure [2–4]. While implantable cardioverter-defibrillators (ICDs) are recommended for the treatment of arrhythmias [5], implantation of a cardiac resynchronization therapy-defibrillator (CRT-D) is appropriate for patients with a high risk of severe arrhythmias or heart failure [6, 7]. A CRT-D is used for patients with cardiac diseases such as CS, dilated cardiomyopathy to restore the conduction abnormalities by coordinating the pump function of the left and right ventricles.

The point of perioperative management of patients with CS is to prevent arrhythmias and worsening of cardiac dysfunction. The key to successful anesthetic management of CS patients is understanding the characteristic features of this disease and implementing the proper settings of the CRT-D device. Currently, case reports about the anesthetic management of patients with CS are limited [8–12]. As a result, this case report aims to describe the perioperative management of a CS patient with an implanted CRT-D.

## Case presentation

**Fig. 1:**
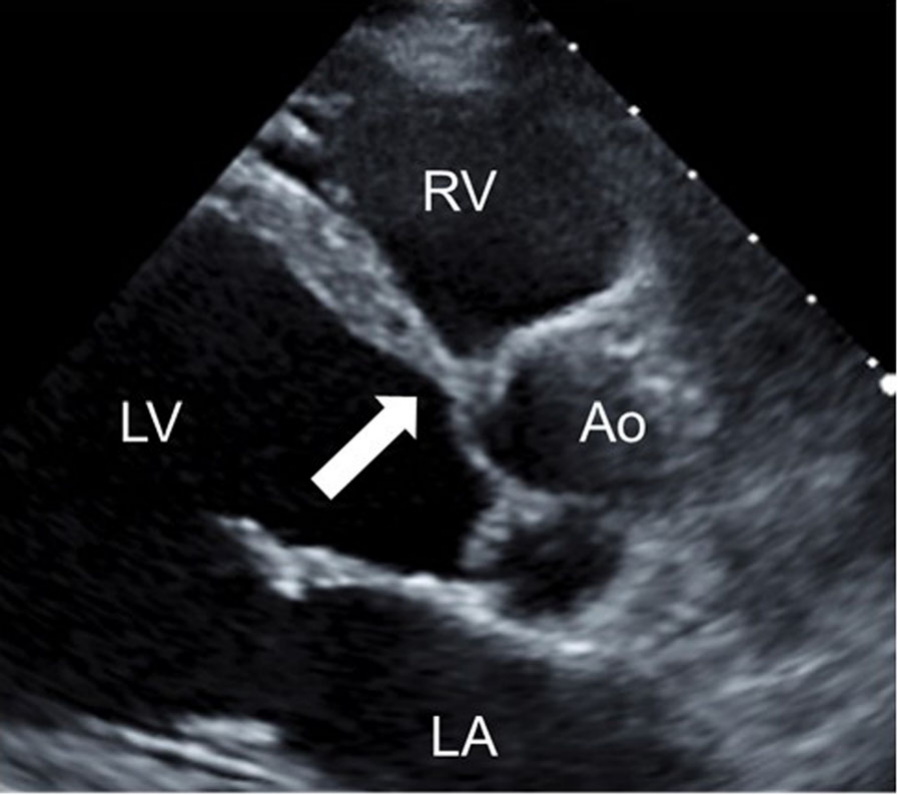
Abnormal thinning of the basal ventricular septum. A parasternal long-axis transthoracic echocardiography view at the level of the aortic valve showing abnormal ventricular septal thinning with a minimal thickness of 4.4 mm (arrow). LA left atrium, LV left ventricle, RA right atrium, RV right ventricle, Ao aorta

A 65-year-old male (height 165 cm, weight 49 kg) was scheduled for a laparoscopy-assisted total proctocolectomy for type 2 colon cancer. His medical history included CS for 2 years and ulcerative colitis for 20 years. The former condition was treated with 5 mg of prednisolone, 2.5 mg of enalapril, 12.5 mg of spironolactone, 5 mg of bisoprolol fumarate, and 50 mg of amiodarone, and the latter with 2400 mg of mesalazine. A CRT-D device was implanted due to symptomatic non-sustained ventricular tachycardia occurring shortly after his CS diagnosis. Before the implantation, the QRS duration was 136 ms and his left ventricular ejection fraction (LVEF) was 30.0% using the modified Simpson method, and 35.2% 4 months after the implantation. Although he experienced no firing of the defibrillator after device introduction, his cardiac function gradually declined to a New York Heart Association (NYHA) class III on admission. The CRT-D device was programmed in the dual-chamber pacing (DDD) modality at a lower rate of 60 beats/min, with the checkup record revealing that 94% of the cardiac rhythm was dependent on the device. The electrocardiogram (ECG) presented a ventricular-paced rhythm with a heart rate of 75 beats/min. Preoperative transthoracic echocardiography showed overall left ventricular wall motion abnormalities with a LVEF of 32.0%. Abnormal ventricular septal thinning, one of the main criteria for CS [13], was also detected (Fig. 1). In addition to CS, a positron emission tomography-computed tomography (PET-CT) showed bilateral hilar lymphadenopathy and several nodules in the lungs indicative of pulmonary sarcoidosis, despite a lack of clinical pulmonary manifestations. Additional examinations, including laboratory testing, a spirogram, and a chest X-ray, were within the normal range.

We selected general anesthesia combined with epidural anesthesia (Th11-12) for perioperative management of this patient. We maintained the usual prednisolone medication on the operation day and administered 100 mg of hydrocortisone pre-operatively. The induction of anesthesia and the tracheal tube intubation were uneventful, using 100 μg of fentanyl, 3 mg of midazolam, and 30 mg of rocuronium. Anesthesia was maintained with sevoflurane (1.0–1.5%) and remifentanil (0.1–0.3 μg/kg/min). In addition to standard monitoring, invasive arterial pressure via the left radial artery, central venous pressure via the right internal jugular vein, stroke volume index, cardiac index, stroke volume variation, systemic vascular resistance, and central venous oxygen saturation were monitored with the use of the FloTrac/PreSep/EV1000^TM^ system (Edwards Lifesciences, Irvine, CA, USA). The modality of the CRT-D was unchanged except for its defibrillation function, which was deactivated before induction of anesthesia to avoid malfunctioning during surgery. Instead, defibrillation pads were placed on the patient and connected to another defibrillator in case of fatal arrhythmias.

**Fig. 2:**
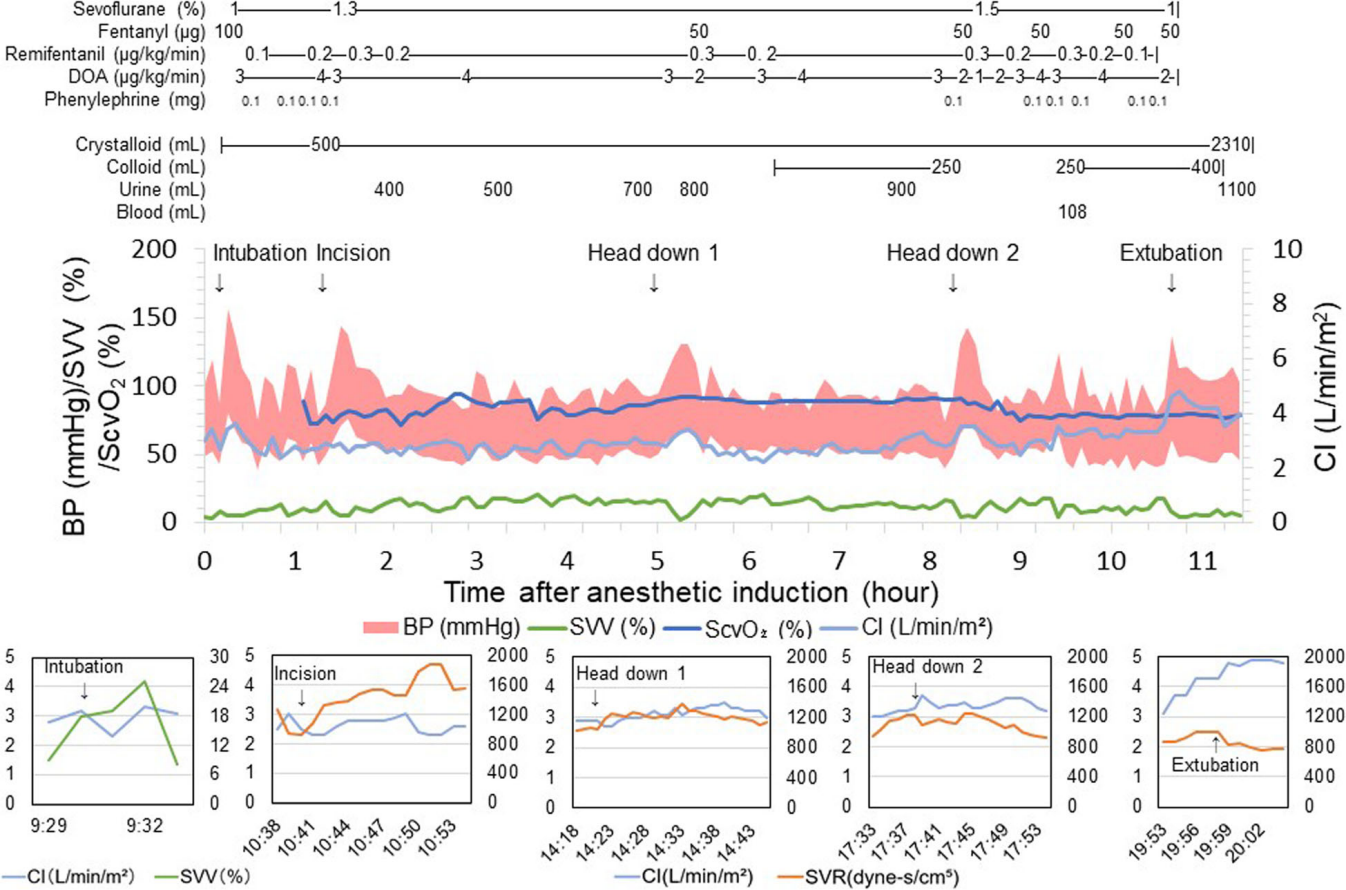
Anesthesia record of a patient with cardiac sarcoidosis who underwent laparoscopy-assisted total proctocolectomy. DOA dopamine, BP blood pressure, SVV stroke volume variation, CI cardiac index, ScvO_2_ central venous oxygen saturation, SVR systemic vascular resistance

As shown in Fig. 2, some hemodynamic fluctuations occurred during the course of the surgery, especially when the patient was intubated, the surgical procedure was started, the pneumoperitoneum was started, and the posture of the patient was changed. However, those changes were successfully managed with bolus administration of phenylephrine, continuous infusion of dopamine, bolus infusion of colloid, and/or titration of anesthetics, in accordance with the abovementioned monitoring. The total operation time was 8 h and 52 min, and the total anesthesia time was 10 h and 47 min. The total infusion volume was 2710 ml, and the total amount of bleeding and urine output were 108 and 1208 ml, respectively. After the surgery, the defibrillation function was reactivated, and the patient was uneventfully extubated. His postoperative pain was successfully controlled with a continuous epidural infusion of 0.125% levobupivacaine (5 ml/h) that was started before the end of the surgery. The intensive cardiac monitoring was continued just before he was transferred from the intensive care unit to the ward, while continuous ECG and SpO_2_ monitoring were performed until postoperative day (POD) 3. He was discharged on POD 24 without any deterioration of his cardiac condition.

## Discussion

As far as we know, this is the first case report describing the anesthetic management of a CS patient with an implanted CRT-D. We accomplished safe anesthetic management by understanding the characteristic features of this disease, implementing the proper settings of the CRT-D device, and preventing progressive heart failure by using the FloTrac^TM^/PreSep/EV1000^TM^ system.

Racial differences in the incidence of CS have been reported, with Japanese patients more frequently reported to have the disease in comparison to Caucasian or African-American patients [2, 14, 15]. Cardiac involvement is reported clinically in 5% of sarcoidosis patients; however, autopsy and cardiovascular magnetic resonance studies have shown a higher percentage of subclinical cardiac involvement, ranging from 15 to 80%, which accounts for the majority of sarcoidosis-related deaths [2, 3, 14]. Clinical management of arrhythmias include the use of antiarrhythmics and/or the placement of an automatic ICD [2, 4]. ICD placement is a class 1 recommendation for CS patients with spontaneous sustained ventricular arrhythmias, a previous cardiac arrest, or a LVEF ≤ 35% despite optimal medical therapy and a period of immunosuppression [5]. CRT placement is also considered for CS patients who are at risk for the development of heart failure [6, 7].

Case reports of anesthetic management for CS patients are limited. In one report, the CS patient had a pacemaker [10]; in another report, the patient had an ICD [11]; and in two other reports, the patients did not have any devices [8, 9]. In another case, emergency introduction of a temporary pacemaker via the jugular vein was needed due to a complete atrioventricular block during surgery [12]. However, we could find no reports related to anesthetic management of CS patients with implanted CRT-Ds.

CRTs are used to correct conduction abnormalities by coordinating the pump functions of the left and right ventricles. However, when comparing a cohort of patients with CS with a cohort of patients with dilated cardiomyopathy, the response to CRT was lower, with a higher rate of major adverse cerebral and cardiovascular events (MACCE) [7]. Moreover, among CS patients, the occurrence of ventricular tachycardia/fibrillation (VT/VF) was elevated [16]. These higher complication rates may be attributable to LV dysfunction due to sarcoid lesions and granulomas, as well as widely spreading fibrosis in the myocardium, which exceeded the benefit of CRT in CS patients [7]. In the present case, the patient’s cardiac function had progressively worsened since CRT-D implantation. Thus, clinicians should be aware that there is still a risk of cardiac compromise, even in the presence of a CRT-D, and especially in perioperative settings where many factors, including the stress response, fluid shifts, and hormonal changes, can have substantial influences on the heart and the whole body. Potential intraoperative problems related to CRT-Ds include electromagnetic interference (EMI) caused by surgical devices and the malfunctioning of the defibrillator. We maintained the DDD pacing mode throughout the operation. Shifting the pacing mode to asynchronous pacing, such as VOO, is useful for preventing EMIs; however, the VOO mode of a CRT-D cannot synchronize the left and right ventricles. In order to maintain cardiac output, it is desirable to continue with the synchronous pacing mode if possible.

Anesthetic strategies for patients with sarcoidosis have not clearly been determined due to a limited amount of published research, and no deteriorative anesthetic agents have been reported. In this patient, we chose to use sevoflurane because it has less of an effect on myocardial contractility [17] and is less likely to cause arrhythmias [18, 19]. Remifentanil, which has a direct negative chronotropic effect [20], was also safely used in this patient, partially due to the support of the CRT. Nevertheless, the long-term effect of anesthetic drugs on patients with sarcoidosis remains unknown, and further research should be conducted in this field. Continuous epidural anesthesia was used because we believed that reducing the postoperative afterload would be effective in preventing worsening of heart failure. On the other hand, we refrained from bolus administration intraoperatively because the reduction of the preload by epidural anesthesia would disrupt the hemodynamics of this patient.

This patient’s laparoscopic surgery required a significant amount of time in the head-down and head-up positions. The head-down position increases the preload, and the head-up position decreases it. In order to avoid excessive preload and afterload, it was helpful to monitor the stroke volume variation (SVV) and the systemic vascular resistance (SVR) using the FloTrac/PreSep/EV1000^TM^ system. Although the usefulness of dynamic parameters such as SVV in laparoscopic surgery are controversial, anesthesia time in this case was more than 10 h, which increased the importance of sequential hemodynamic monitoring for this patient.

In this patient, the only clinical manifestations of sarcoidosis were in the heart. However, clinicians should be wary about inherent limitations in the sarcoidosis diagnosis, including clinically undetectable inflammation in other organs or cardiac lesions that arise only as an original manifestation [14]. Thus, clinicians should be careful to examine all organs that can be affected by sarcoidosis. Indeed, although this patient did not show any symptoms, the PET-CT demonstrated lung involvement of the sarcoidosis, which can induce both a restrictive and an obstructive pulmonary disorder with wheezing and bronchial hyperreactivity, even in the absence of evident morphological abnormalities of the tracheobronchial tree [21]. Sarcoidosis of the upper respiratory tract, including laryngeal areas, should also be considered because it can occur in up to 5% of patients [22] and can cause difficult intubations, airway obstruction, and post-extubation croup.

We achieved safe anesthetic management of a patient with cardiac dysfunction caused by CS, who was previously implanted with a CRT-D. Although intraoperative cardiovascular system monitoring contributed to the successful anesthetic management, a detailed understanding of this patient’s condition and his sarcoidosis diagnosis was indispensable for the proper perioperative management of this patient.

## Data Availability

Data relevant to this case report are not available for public access because of patient privacy concerns but are available from the corresponding author on reasonable request.

## Abbreviations

CS: Cardiac sarcoidosis
CRT-D: Cardiac resynchronization therapy-defibrillator
DDD: Dual-chamber pacing
ICD: Implantable cardioverter-defibrillator
NYHA: New York Heart Association
ECG: Electrocardiogram
LVEF: Left ventricular ejection fraction
PET-CT: Positron emission tomography-computed tomography
POD: Postoperative day
MACCE: Major adverse cerebral and cardiovascular events
VT/VF: Ventricular tachycardia/fibrillation
